# Co-Digestion of Sugarcane Biorefinery Byproducts in Upflow Anaerobic Sludge Blanket Reactors, in Series, with Application of High Organic Loading Rate

**DOI:** 10.1021/acsomega.5c06244

**Published:** 2026-08-21

**Authors:** Bruna R. S. Sturaro, Lucas A. L. de Carvalho, Daniel G. Pinheiro, Roberto A. de Oliveira, Rose M. Duda

**Affiliations:** † Graduate Program in Agricultural and Livestock Microbiology, School of Agricultural and Veterinary Sciences, São Paulo State University (UNESP), Jaboticabal, São Paulo 14884-900, Brazil; ‡ Technology Faculty “Nilo de Stéfani”, Paula Souza Center, Jaboticabal, SP, Brazil, Av. Eduardo Carlos Zambianchi, n. 31, Vila Industrial, Jaboticabal, São Paulo 14883-130, Brazil; § Graduate Program in Agricultural and Livestock Microbiology, Engineering Department, School of Agricultural and Veterinary Sciences, São Paulo State University (UNESP), Jaboticabal, São Paulo 14884-900, Brazil

## Abstract

Anaerobic digestion of products from the sugarcane industry is an interesting option for biogas production. In this work, the anaerobic digestion of vinasse and sugarcane molasses supplemented with filter cake was performed in a two-stage upflow anaerobic sludge blanket (UASB) reactors system (R1 and R2). A volumetric methane production (VMP) of up to 4.3 L CH_4_ (L d)^−1^ was obtained for R1 + R2, with the application of an organic loading rate (OLR) in R1 of up to 45.0 g total COD (L d)^−1^. However, for OLR values higher than those applied, a shock load was observed, and only after its reduction was it possible to obtain stability again in terms of the pH, alkalinity, and acidity values in the UASB reactors, R1 and R2, which highlights the robustness of these anaerobic systems. With the decrease in the OLR after the shock load, the genera *Trichococcus* and *Methanosarcina* prevailed over *Bifidobacterium* and *Methanobacterium* in the sludge of the UASB reactor (R1). The use of two-stage UASB reactors for the co-digestion of vinasse, molasses, and filter cake allowed the establishment of a maximum OLR for the operation of anaerobic reactors under stable conditions in terms of the production of biogas and quality effluent. This could help the sugar and energy industries to produce methane in a sustainable and optimized manner.

## Graphical abstract

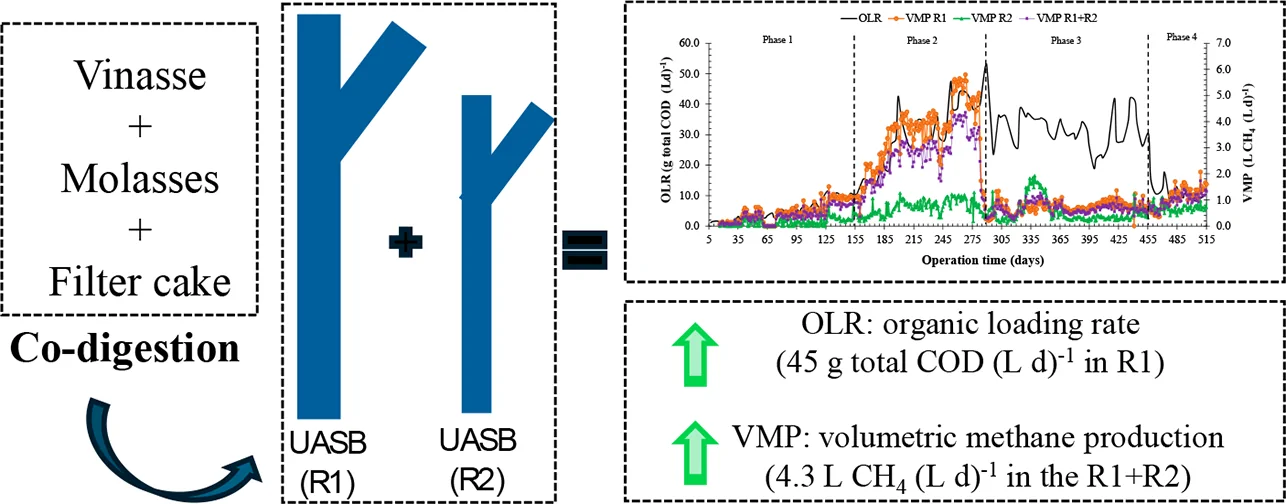

## 1 Introduction

The increase in the world population and the development of industries in recent decades have increased energy consumption.^1^ Environmental concerns and recent technological advances have made renewable energy an option for expanding power generation capacity, with sources including ethanol and biogas. Electricity generation from renewable sources increased from 22.7% in 2015 to 32.1% in 2024.^2^ Awareness of environmental impact has motivated a growing emphasis on global policies that drive the transition to the use of biofuels and the reduction of emissions.^3^ This change has occurred because of concerns about the effects of energy from fossil fuels, whose potential to contribute to pollution, climate change, and natural disasters has been widely recognized.^1^

The amount of sugarcane ethanol produced in Brazil in the 2024/2025 harvest was approximately 37.2 billion liters.^4^ Concerns about the byproducts of the Brazilian sugarcane industry, especially vinasse, are important because of the volume produced. For each liter of sugarcane ethanol produced, 10 to 14 L of vinasse are generated^5,6^ and have a high organic load because of the presence of phenolic compounds, glycerol, ethanol, melanoidins, sugars, volatile fatty acids, and others^7^ and are currently used in the fertigation of sugarcane fields.^8^

Anaerobic digestion of vinasse has been recognized as an alternative for vinasse treatment and biogas production. The removal of biodegradable organic matter in anaerobic reactors reduces methane emissions during the storage and transportation of treated vinasse, and the land application of biodigested vinasse reduces nitrous oxide emissions by 78% compared with te application of untreated vinasse^9^ and can also be used in the fertigation of sugarcane fields to take advantage of nutrients and water. The nutrients contained in vinasse are generally preserved by anaerobic digestion.^7^

Despite the advances indicated in the anaerobic digestion of vinasse, studies are still needed to optimize the stability of biogas production.^5,10,11^ Some alternatives for increasing biogas production have been demonstrated from the anaerobic co-digestion of vinasse and other byproducts of the sugar-energy industry, such as filter cake^5^ and bagasse.^12^

Co-digestion is a technique that involves mixing different types of organic waste in the same anaerobic reactor, with the aim of improving biogas production and increasing treatment efficiency. For co-digestion, it is important to select the organic waste that will be mixed according to its physical-chemical characteristics and availability in the region. This technique is promising for improving biogas production in the sugar-energy industry, contributing to more sustainable waste management and reducing the environmental impact.^13^

Molasses, filter cake, and vinasse can be interesting sources of raw material to produce biogas from byproducts of the sugar-energy industry. Molasses is a byproduct of sugar production with a density between 1.4 and 1.5 g mL^–1^ and a productivity of 40 kg ton^–1^ of sugarcane.^14^ The composition of sugarcane molasses is highly variable, as it depends on agricultural and industrial factors. The main components of molasses are water (17 to 25%), sucrose (30 to 40%), fructose (5 to 12%), glucose (4 to 9%), ash (7 to 15%), other carbohydrates, proteins (2.5 to 4.5%), and compounds of organic origin such as amino acids, carboxylic acids, vitamins, phenols, and others.^15^

Anaerobic digestion of vinasse and molasses has been reported in several studies as an interesting strategy for biogas production.^5,9,16,17^ Molasses can also be an alternative for the maintenance of anaerobic reactors during the sugarcane off-season, as it can replace vinasse.^18,19^ Several studies have investigated the co-digestion of different proportions of vinasse and sugarcane molasses using different anaerobic reactor configurations, temperature ranges, and chemicals to correct the pH of the influent.^17,20–22^ Studies are needed to identify the OLR limits for stable maintenance of biogas production in UASB reactors co-digesting vinasse and molasses, without the need for chemicals to correct the pH of the influent. The use of alkalizing agents increases the operating costs of anaerobic reactors in the co-digestion of vinasse and sugarcane molasses, and excess sodium bicarbonate, for example, can lead to sodium toxicity.^17^

Co-digestion of vinasse and molasses can be facilitated if there is a nutritional balance, since nutrient availability is an important factor in anaerobic treatment because of the high concentrations of organic material in vinasse and molasses. Normally, the concentrations of nitrogen (N) and phosphorus (P) in vinasse do not meet the recommended amounts for optimal anaerobic digestion.^5,16^ For biomass with a high yield coefficient (*Y* ∼0.15 g VSS/gCOD), e.g., degradation of carbohydrates, the recommended value is a COD/N/P = 350:5:1.^23^ Co-digestion of vinasse and sugarcane molasses could reduce process inhibition by promoting mixing and dilution of the toxic compounds present in vinasse and the high levels of salts and nitrogen in molasses.^17^

The most technically and economically appropriate solutions for the anaerobic treatment of industrial waste should be explored with nutrient supplementation.^24^ Filter cake, a byproduct of the sugar-energy industry, is an interesting alternative that can reduce costs by eliminating the use of industrialized chemical compounds in anaerobic digestion. Filter cake is rich in nutrients, with N and P concentrations of 1.72 and 0.6% of total solids (TS), respectively,^16^ and K, Ca, Mg, and S concentrations of 0.3, 2.1, 0.6, and 0.25% on a dry weight basis, respectively.^25^ Filter cake is currently used as an energy source in the production of steam for electricity generation and as a fertilizer in agriculture.^26^

Among the various anaerobic reactor configurations, upflow anaerobic sludge blanket (UASB) reactors are the most popular for the anaerobic digestion of vinasse because of the possibility of applying low hydraulic detention times. However, the stability of biogas production is a typical problem in full-scale applications.^11^ A UASB reactor is a biological treatment system that uses the action of microorganisms in an oxygen-free environment to remove organic matter from vinasse and produce biogas.^27^ A representative example of the full-scale application of UASB technology is a 5,000 m^3^ reactor located in the Ribeirão Preto region, São Paulo State, Brazil. The reactor was operated at 55 °C to produce biogas from sugarcane vinasse without nutrient supplementation. Under these operating conditions, treatment efficiency was considered modest because the primary objective was the generation of sufficient biogas to supply the energy demand for yeast drying.^28^ In other words, economically viable strategies for the use of byproducts from the sugar-energy industry are needed.

The objective of this work was to evaluate the maximum allowed OLR for the stable performance of two UASB reactors (R1 and R2) in series in the co-digestion of vinasse and molasses, 50%/50% (%COD/%COD) supplemented with filter cake, with respect to biogas production and effluent quality. The application of this type of system, with an applied OLR, for the recovery of vinasse, molasses, and filter cake resources is unprecedented and provides extremely relevant information. It helps the sugar and energy industries apply this large-scale reactor project to produce methane in a sustainable way, contributing to important issues of new collective awareness involving environmental, social, and governance aspects.

## 2 Materials and Methods

### 2.1 Experimental Structure

The experimental unit consisted of an influent and effluent storage tank, a diaphragm pump, followed by two UASB reactors in series, with volumes of 12.1 L (first stage—R1) and 5.6 L (second stage—R2), and two fiberglass gasometers^5^ (Figure 1).

**Figure 1:**
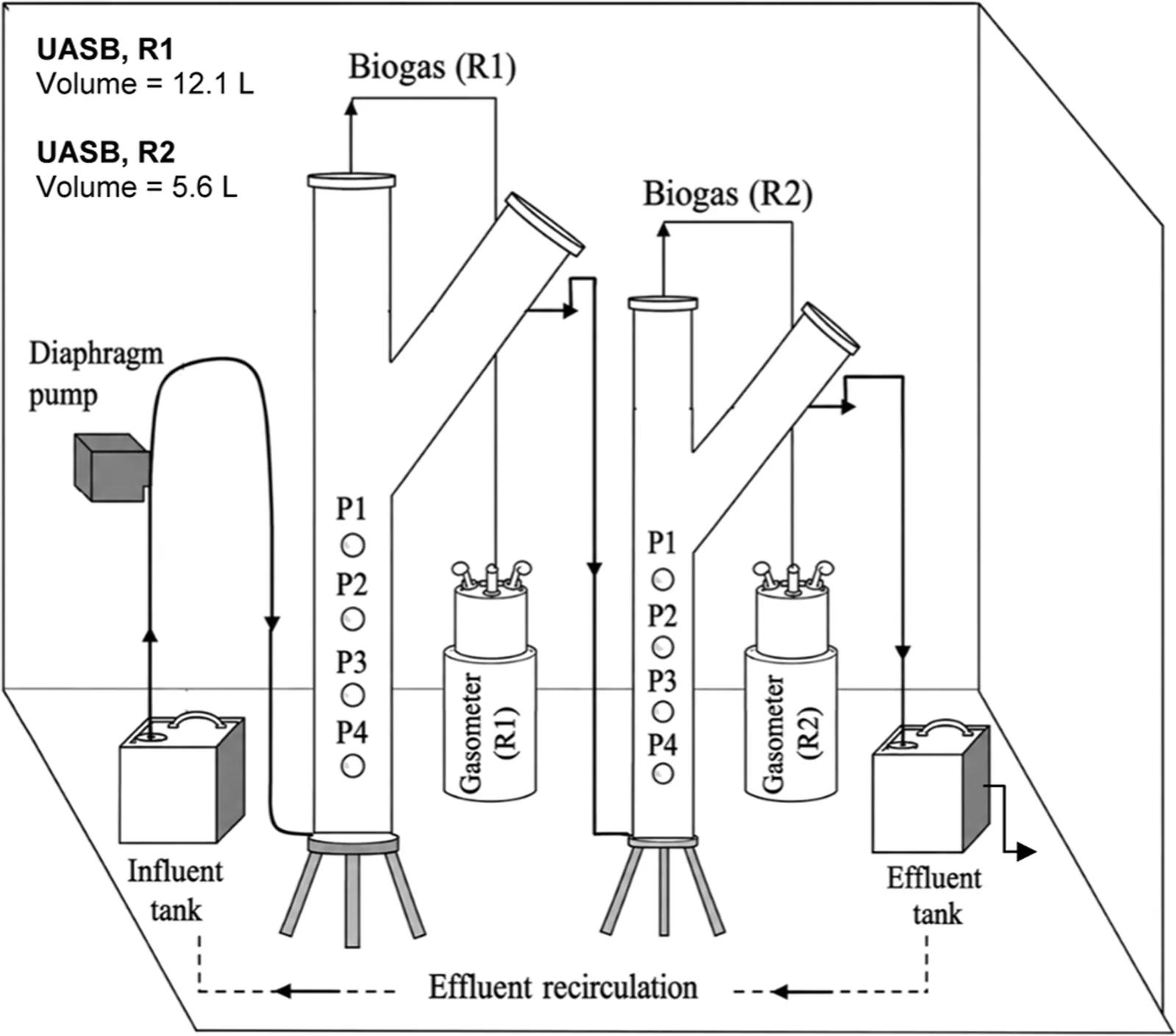
Schematic of two upflow anaerobic sludge blanket reactors (UASB, R1 and R2) in series with unconventional Y-shaped gas separators. P1–P4 are points to collect sludge.

The reactors were built with PVC pipes, 100 and 75 mm in diameter, for R1 and R2, respectively, with Y-shaped phase separators at an angle of 45° to the vertical,^29^ as adapted by other studies.^24^ Each reactor was distributed along its height, with four points for collecting sludge samples, P1, P2, P3, and P4, with 25 cm between each point. The UASB reactors were kept in a climate-controlled chamber with a temperature control system (35 °C), which corresponds to the optimal mesophilic temperature for anaerobic digestion.^23^

### 2.2 Influent and Inoculum Sludge Used

The vinasse was collected in the first distillation column in a sugarcane industry located in the Ribeirão Preto region of São Paulo State, Brazil.

The filter cake was collected at the outlet of the rotary filters of the same agroindustry where the vinasse was collected. These filters remove impurities and particles in the production of sugar, retaining solid residues in the filter cake after the filtration of the sugarcane juice.

The molasses used was molasses B obtained at the end of the previous harvest (84.7% soluble solids) and stored at room temperature. The combination of molasses with vinasse in anaerobic co-digestion is advantageous because it provides extra nutrients for the microorganisms in the process. Molasses, which is rich in carbohydrates and nutrients, complements vinasse, which contains complex organic materials. This balanced mixture optimizes biogas production, increasing the efficiency of the energy generation. This cooperation between both materials provides an environment for the growth of methanogenic microorganisms, improving the overall methane-production process.

The inoculum sludge used in the reactors was obtained from the anaerobic treatment of vinasse and the filter cake. The characteristics of the inoculum sludge used in the UASB reactors were identical, with a total solid (TS) concentration of 46.91 g L^–1^ and a volatile solid (VS) concentration of 28.52 g L^–1^. The volume of inoculum sludge placed in the UASB reactors was sufficient to meet the organic loading rate in the sludge of 0.05 g total COD/g VSS.d,^23^ which corresponded to approximately 30% of the volume of the UASB reactors (R1 and R2).

### 2.3 Operation of the Treatment System

The hydraulic detention times (HDTs) applied were 24 h for the first stage (R1) and 11.1 h for the second stage (R2) (Table 1).

**Table 1:** Operating Conditions of UASB Reactors in Series (R1 and R2), Used in the Anaerobic Co-Digestion of Vinasse and Molasses Supplemented with Filter Cake, in Phases 1 to 4^a^

	R1	R2
Volume (L)	12.1	5.6
HDT (h)	24.0	11.1

	Phase 1	Phase 2	Phase 3	Phase 4	Phase 1	Phase 2	Phase 3	Phase 4
OLR	4.8	29.5	31.7	11.0	5.0	17.6	47.2	16.2
VC	65	40	20	25	72	72	25	39

a UASB: upflow anaerobic sludge blanket reactor; HDT: hydraulic detention time; OLR: organic loading rate in g total COD (L d)^−1^; total COD: total chemical oxygen demand. VC: variation coefficient in %.

Anaerobic co-digestion of vinasse (V) and sugarcane molasses (M) was performed using a 50% V/50% M ratio in terms of total chemical oxygen demand. To gradually increase the OLR, the amounts of vinasse and molasses were also increased in the influent, always using a ratio of 50%/50% (%COD/%COD). The amounts used varied from 0.22 to 8.20 L of vinasse, 0.05 to 0.16 L of molasses, and from 3.75 to 11.83 L of recirculated effluent from R2.

The average values of OLRs applied to the UASB reactors (R1) and (R2) were 4.8, 29.5, and 31.7 g total COD (L d)^−1^ and 5.0, 17.6, and 47.2 g total COD (L d) ^–1^, in phases 1, 2, and 3, respectively. The OLR in R1 gradually increased to values close to 48 g total COD (L d)^−1^ in phase 2.

In phase 2, when the OLR was close to 50 and 100 g total COD (L d)^−1^ in R1 and R2, respectively, a load shock was observed in the reactors. This required the use of some strategies, such as removing recirculation and correcting the influent pH to values close to 7.0 with a calcium hydroxide solution (56 g L^–1^). Recirculation was not used in the periods from 289 to 299, from 334 to 343, and from 378 to 420 days of operation. Calcium hydroxide was used to correct the influent pH in the periods from 288 to 293 and from 350 to 450 days of operation. Even with these strategies, it was not possible to stabilize the system. Therefore, the OLR applied in R1 was reduced to values close to 11.0 g total COD (L d)^−1^ (Phase 4), which allowed the reduction in total volatile acids and the total alkalinity ratio in the UASB, R1 and R2 reactors.

The recirculation rate was 0.98 to 0.35 in phases 1 to 4, respectively, as described by Zuo et al.^30^

### 2.4 Supplementation with Filter Cake in the Influent

The filter cake was collected wet in the industry. However, for storage and addition to the influent, the filter cake was dried naturally simply by exposure to open air. The filter cake was used for phosphorus (total-P) supplementation of the influent, on the basis of the COD/P ratio of 350:1 recommended for anaerobic digestion.^23^ The COD/P ratio of the vinasse used as influent in UASB reactors operated by Santana Junior et al.^18^ ranged from 350:0.4 to 350:0.3, and the diluted molasses ratio was 350:0.6, indicating the need for supplementation. In addition, the filter cake also contains 9.04, 0.125, 0.312, and 0.018 mg g^–1^ TS of total-P, Cu, Mn, and Fe, respectively.^5^ To add the filter cake to the influent, a solution composed of filter cake + molasses + vinasse + R2 effluent was prepared. Approximately 1 g of filter cake was added for every 1000 g of COD added.^5^ The solution was allowed to stand for 24 h. After this period, the influent was sieved through a 2 mm mesh sieve and subsequently filtered through cotton fabric to separate the fibers present in the filter cake and avoid clogging the pump and pipes.

The preparation and addition of filter cake in full-scale systems can be easily achieved with industrial equipment such as sieves and filtration systems, which are common in the sugar and energy industries. The fibers and other materials from the filter cake retained during filtration can be sent to the field with excess filter cake produced in the industry.

### 2.5 Monitoring of the System: UASB Reactors in Series

Table 2 presents the physical examinations and chemical determinations performed on composite samples of influents and effluents, sludge, and biogas during monitoring of UASB reactors, as well as the frequencies and sources of the methodologies.

**Table 2:** Examinations and Determinations on Samples, Frequency, and Methodologies Used

Examinations and determinations	Frequency	References
Influent and effluent		
pH	daily	Method: 4500H + B^31^
Total chemical oxygen demand (total COD) and dissolved chemical oxygen demand (diss COD)	twice\week	Method: 5220 – B^31^
Total (TA), partial (PA), and intermediate (IA) alkalinity	daily	Jenkins et al.^32^
Total volatile acids (TVA)	twice\week	DiLallo and Albertson^33^
Kjeldahl Nitrogen (KN)	once\week	Method: 4500-NC^31^
Ammoniacal nitrogen (Am-N)	twice\week	Method: 4500-NH_3_ B^31^
Total phosphorus (total-P)	once\week	Method: 4500-PC^31^
Total suspended solids (TSS) and volatile suspended solids (VSS)	once\week	Methods: 2540 – C and 2540 – E^31^
Sulfate and sulfide	once\week	Methods: 4500-SO4 2 E and 4500-S2 F^31^
Fe, Cu, Zn, Mn, Ca, Mg, Na, K, Ni, and Co	fortnightly	Method: 3030 – H and 3111 – A^31^
Sludge		
Total solids (TS) and volatile solids (VS)	end of trials 1, 3, and 4	Method: 2540 – B and 2540 – E^31^
Biogas		
Production	daily	Gasometers^24^
Composition	fortnightly	Method: 2720 C^31^

Biogas production was measured daily using fiberglass gasometers as described by Villa-Montoya et al.^24^ The composition was determined biweekly by gas chromatography (Thermo Scientific) using a Q Poropak column, H_2_ as the carrier gas, and a thermal conductivity detector. The estimated electrical energy production (En) was calculated according to Kythreotou et al.,^34^ considering a generator efficiency of 35%, a methane density of 0.6556 kg m^–3^, and a higher heating value of methane (55.6 MJ kg^–1^). To estimate the energy required for sugarcane processing, 28 kWh of electrical energy is needed to process each ton of sugarcane,^3^ and one ton of sugarcane can generate, on average, one cubic meter of vinasse.^18^

### 2.6 Statistical Analysis

Statistical analyses of the mean values obtained were performed using the F test and Tukey’s 5% test, considering a completely randomized design, with four treatments (Phases 1, 2, 3, and 4) for the UASB reactors, R1 and R2, with repetitions in time for each attribute evaluated. The analyses were performed using Sisvar software. The microbial community data were excluded from the above statistical framework, as only a single sample was sequenced by experimental condition (*n* = 1), precluding inferential analysis; therefore, all microbiome-related results are presented and discussed descriptively throughout the manuscript.

### 2.7 Microbial Community Analysis

#### 2.7.1 DNA Extraction

Samples for genomic DNA extraction and sequencing were collected from the inoculum sludge of the reactors and from the four sludge collection points distributed along the UASB reactors in series on days 348 and 505 of operation, corresponding to phases 3 and 4, respectively. The sludge samples from the collection points of each reactor were homogenized to obtain representative samples from the reactors R1 and R2.

DNA extraction and quality assessment were performed by the sequencing facility (ByMyCell, Ribeirão Preto, São Paulo, Brazil) using the ZymoBIOMICS DNA Miniprep Kit (Zymo Research, Irvine, CA, USA) following the manufacturer’s instructions. DNA quantity and integrity were evaluated prior to library preparation, and all samples met the facility’s minimum quality thresholds for downstream processing.

#### 2.7.2 Sequencing on the Illumina Platform

The V4 hypervariable region of the 16S rRNA gene was amplified using the primer pair 515F (5′-GTGCCAGCMGCCGCGGTAA-3′) and 806R (5′-GGACTACHVGGGTWTCTAAT-3′).^35^ Library preparation, including PCR amplification, indexing, and pooling, was conducted at the ByMyCell facility. Sequencing was performed on the Illumina MiSeq platform (2 × 250 bp paired-end layout), generating a total of 531,066 raw reads across the five samples (approximately 106,213 reads per sample).

#### 2.7.3 Data Analysis

The initial quality of the raw sequencing reads was assessed by using FastQC (v0.11.9). Adapter removal and quality filtering were performed with Fastp (v0.23.2 ^36^), discarding reads with an average Phred quality score of less than 25 (-average_qual = 25). Forward and reverse reads were merged using FLASH (v1.2.11 ^37^) with a minimum overlap of 10 bp (--min-overlap = 10). Only merged reads with a final length of greater than 435 bp were retained for downstream analysis.

Merged reads were processed using the DADA2 pipeline,^38^ implemented in the dada2 R package within the R environment (v4.1.2 ^39^). Quality filtering was applied using the filter and Trim function with a maximum expected error threshold of 3 (max EE = 3). Base-specific error rates were estimated using the learn Errors function, and reads were denoised using the inferred error model via the dada function. Amplicon sequence variants (ASVs) were identified, and chimeric sequences were removed using the Bim era Denovo function. Singleton ASVs are inherently excluded by the DADA2 denoising algorithm, which requires consistent evidence across reads to call a true sequence variant.^38^ After quality filtering, merging, and chimera removal, a total of 518,774 reads were retained across the five samples (mean: 103,755 reads per sample; range: 100,253–110,776).

Taxonomic classification of ASVs was performed using the assign Taxonomy function in DADA2, which implements the Naïve Bayesian classifier method,^40^ against the SILVA reference database (v138 ^41^). Classification was performed with default parameters, including a bootstrap confidence threshold of 50% (min Boot = 50) over 100 bootstrap iterations. ASVs not classified within the Bacteria or Archaea domains or those annotated as chloroplasts or mitochondria were excluded from the data set. After filtering, a total of 1465 ASVs were retained across the five samples.

ASV count tables and taxonomic assignments were combined into a phyloseq object using the phyloseq R package (v1.38.0 ^42^). For compositional analyses, absolute ASV counts were transformed into relative abundances using the phyloseq_standardize_otu_abundance function from the metagMisc package (v0.04).

Sampling sufficiency was evaluated through rarefaction curves generated with the amp_rarecurve function of the ampvis2 R package (v2.7.17 ^43^). Alpha diversity was estimated by computing the observed richness (number of ASVs) and the Shannon diversity index using the alpha function from the microbiome R package (v1.16.0). Given that a single composite sample was analyzed per condition (*n* = 1), alpha diversity values are reported descriptively, and no statistical comparisons were performed between conditions. Beta diversity was assessed by computing the Bray–Curtis dissimilarity among samples and was visualized through principal coordinate analysis (PCoA) using the ordinate function in the phyloseq package. The ordination is presented for exploratory visualization of compositional distances among samples; no formal statistical test of group separation (e.g., PERMANOVA) was applied, as the absence of biological replicates (*n* = 1 per condition) precludes meaningful hypothesis testing.

The functional potential of the microbial communities was inferred using PICRUSt2 (v2.5.2 ^44^) to predict the metagenomic content in terms of KEGG ortholog (KO) abundance per sample. Prokaryotic ecological and biogeochemical functions were additionally predicted using FAPROTAX (v1.2.7 ^45^), which maps taxonomic profiles to putative functional groups on the basis of the published literature of cultured representatives. Notably, both PICRUSt2 and FAPROTAX generate predicted rather than empirically measured functional profiles. Accordingly, all functional inferences derived from these tools are treated as hypothesis-generating and are interpreted in conjunction with rather than as substitutes for the process performance data obtained in this study.

## 3 Results and Discussion

### 3.1 pH, Alkalinity, and Total Volatile Organic Acids

The pH values of the effluents from the UASB, R1 and R2 reactors, were higher than those of the influent in phases 1, 2, and 4 (Figure 2B), even with the increase in the OLR to values above 45 g total COD (L d)^−1^ , in the R1, of phase 2 (Figure 2A), indicating the buffering capacity of the system for these operating conditions. The recirculation of the R2 effluent also contributed to the increase in partial alkalinity (PA) and pH, without the need for chemicals, in phases 1, 2, and 4.

**Figure 2:**
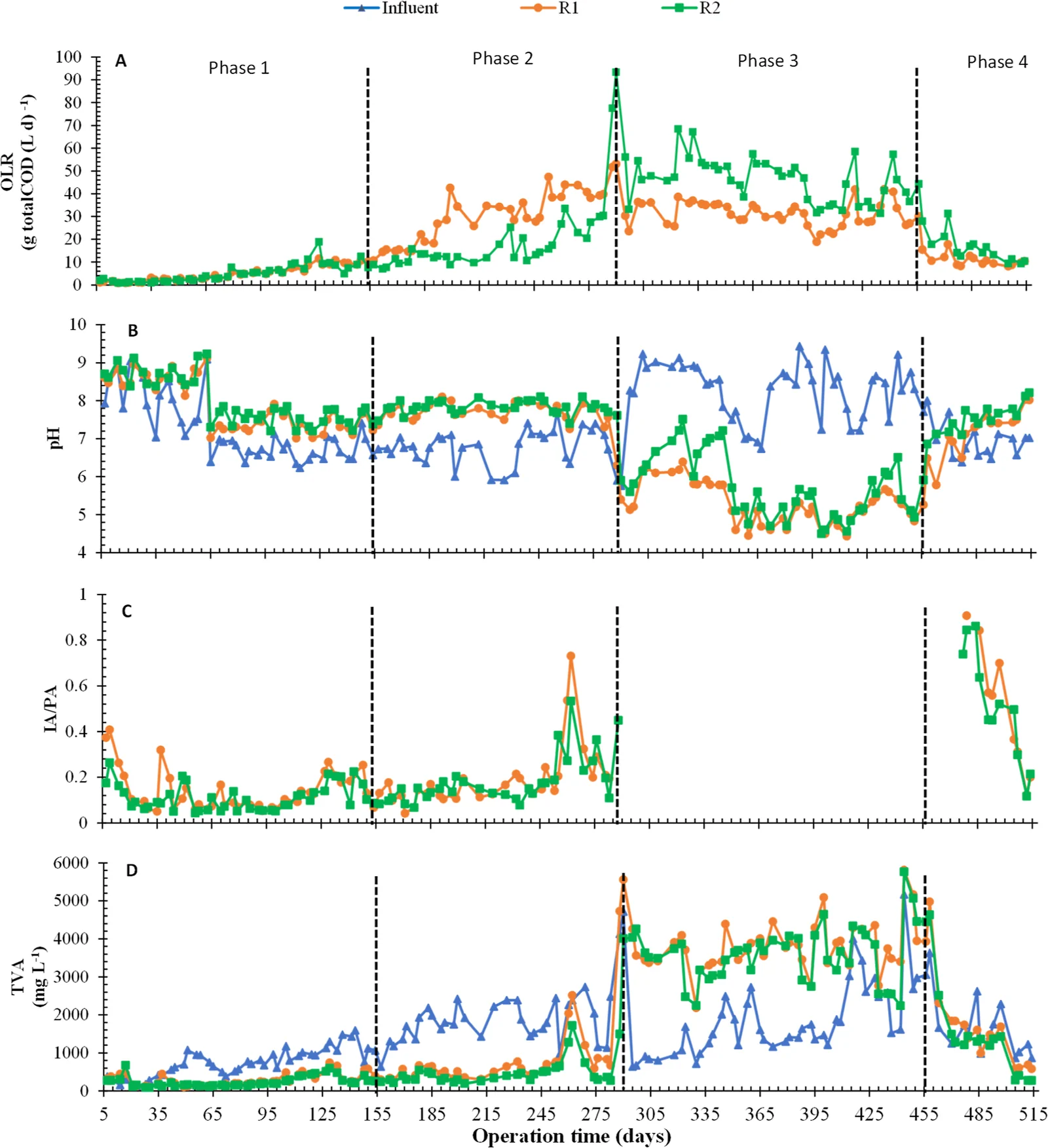
(A) Values of the organic loading rate (OLR), (B) pH, (C) ratio of intermediate alkalinity (IA) to partial alkalinity (PA), and (D) total volatile acids (TVA) in the influent and effluents of the UASB reactors, in series (R1 and R2), in the co-digestion of vinasse with molasses supplemented with filter cake in phases 1, 2, 3, and 4.

The contribution of recirculation to the buffering of the anaerobic system was also reported by Barros et al.,^5^ Caraça et al.,^46^ and Lima et al.,^47^ who treated vinasse and filter cake; vinasse and wastewater from dairy farming; and vinasse and sludge from a water treatment plant. Effluent recirculation also contributed to the dilution of the influent and the adequacy of the OLR.

The pH values were higher than 6.99 in the influent of the UASB, R1 and R2 reactors (Figure 2B) in phases 1, 2, and 4. In the effluent of the UASB, R1 and R2 reactors, pH values close to 9.0 were observed during the first 65 days of operation, and subsequently, a decrease to values close to 8.0 occurred in phases 1 and 2. For the multiplication of microorganisms in anaerobic reactors to occur, the pH must remain between 6.0 and 8.0 ^48^ and in the ideal range of pH 6.8 to 7.4.^49^ However, even with values higher than those recommended, stability was observed in the process, with methane production, in phases 1 and 2. pH values higher than 8.0 were also observed by Barros et al.,^5^ when vinasse was treated in UASB reactors supplemented with filter cake and Caraça et al.,^46^ and when vinasse and dairy cattle manure were treated in UASB reactors.

In phase 3, owing to the load shock (Figure 2A), the OLR reduction strategy was used since the effluent pH values were lower than those observed in the influent (Figure 2B), even with the removal of recirculation and pH correction with calcium hydroxide from the influent. The average values of total alkalinity (TA) and partial alkalinity (PA) increased from the influent to the effluents of the UASB reactors, R1 and R2, in phases 1, 2, and 4 (Table 3).

**Table 3:** Average Values of Partial Alkalinity (PA), Total Alkalinity (TA), Ratio of Intermediary and Partial Alkalinities (IA/PA), and Ratio of Total Volatile Acids and Total Alkalinity (TVA/TA) in the Influent and Effluents of the Upflow Anaerobic Sludge Blanket (UASB) reactors, in series (R1 and R2), Used in the Co-Digestion of Vinasse and Molasses Supplemented with Filter Cake, in Phases 1, 2, 3, and 4^a^

			Phases									
Parameter	Sample		1		2		3		4		VC*	F
PA	influent	average	1295	a	1709	a	1570	a	1605	a	47	ns
		VC	46		46		4		66			
	R1	average	1890	b	4053	a	89	c	2156	b	41	**
		VC	39		22		224		60			
	R2	average	1822	b	4027	a	228	c	2294	b	35	**
		VC	38		16		152		45			
TA	influent	average	1805	b	4085	a	4823	a	4689	a	39	**
		VC	46		43		33		36			
	R1	average	2139	b	4938	a	2214	b	3858	a	34	**
		VC	38		16		55		25			
	R2	average	2150	c	4733	a	2656	c	3862	b	35	**
		VC	44		18		46		22			
IA/PA	R1	average	0.14	c	0.47	c	35.0	a	4.63	b	99	**
		VC	63		351		149		285			
	R2	average	0.13	c	0.17	c	8.84	a	1.13	b	53	**
		VC	128		62		141		285			
TVA/TA	R1	average	0.16	b	0.15	b	2.83	a	0.52	b	138	**
		VC	72		101		87		92			
	R2	average	0.13	b	0.11	b	1.82	a	0.44	a	123	**
		VC	79		105		80		101			

a Units: PA; TA and IA: mg CaCO_3_ L^–1^; VC: variation coefficient in % of each phase. Different lowercase letters in the same line differ according to Tukey’s test at 5%. F - F test; **—Significant at 1% probability (*p* < 0.01); ns—not significant (*p* > 0.05); *p*—probability. VC*—variation coefficient of each parameters for all phases.

The increases in alkalinity in the reactors compared with the influent indicate that there was a rise in partial alkalinity (PA), providing buffering capacity in the reactors. This occurred because of the increase in the bicarbonate concentration, as can be observed through the sharply higher values in the average PA values of the influent for the R1 and R2 effluents (Table 3). The PA values are within the recommended range in the UASB, R1 and R2 reactors, in phases 1, 2, and 4, to maintain a stable anaerobic digestion process, from 1000 to 3000 mg CaCO_3_ L^–1^,^50^ indicating the buffering of the anaerobic system, even when a high OLR is applied.

In phase 3, with an increase in the OLR to average values of 31.7 and 47.2 g total COD (L d)^−1^ (Table 1), respectively, in R1 and R2, reactors decreases in the TA and PA of the influent to the effluent were observed. This occurred because the rise in the OLR reached values of up to 50 and 100 g total COD (L d)^−1^ (Figure 2A) in R1 and R2, respectively, which caused a load shock, resulting in increases in the total volatile acid concentrations (TVAs) to up to 6000 mg L^–1^ in the reactors (Figure 2D) and a decrease in volumetric methane production (Figure 3). No significant differences were observed in the PA of the influent (Table 3), even with the shock load observed in phase 3, which occurred because of the addition of calcium hydroxide to the influent to correct the pH in this phase.

**Figure 3:**
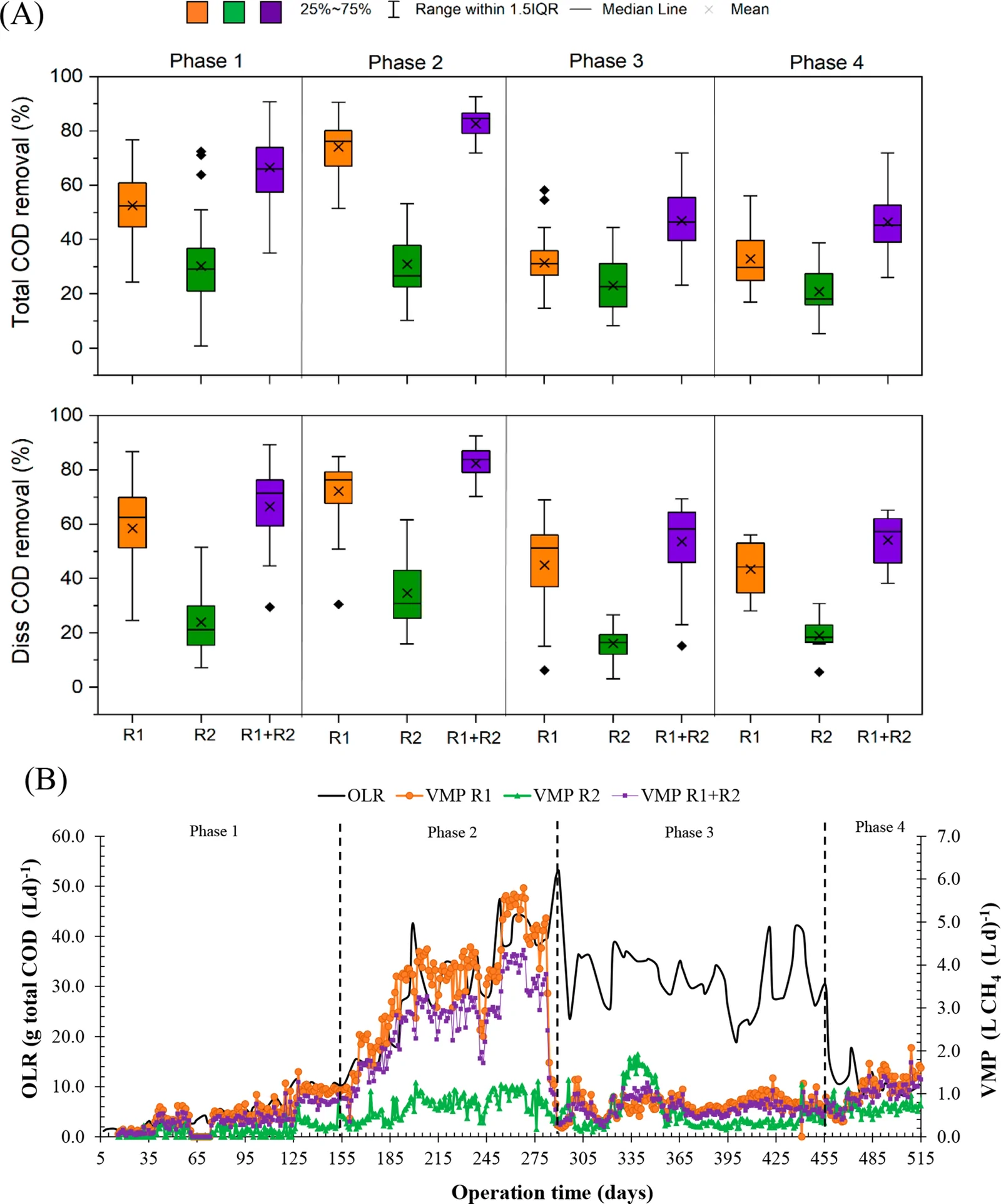
(A) Removal of total chemical oxygen demand (total COD), dissolved chemical oxygen demand (diss COD), and (B) organic loading rate (OLR) applied to the upflow anaerobic sludge blanket reactor (UASB – R1) and volumetric methane production (VMP) in the UASB, R1 and R2 reactors, in series, for the co-digestion of vinasse and molasses supplemented with filter cake in phases 1, 2, 3, and 4. lQR: Interquartile Range.

The ratios between IA and PA reached 0.90 in phases 1, 2, and 4 (Figure 2C). In phase 3, during the period from 351 to 423 days of operation, no PAs were observed for R1 or R2, and during the other periods of this phase, the values were lower than 650 mg L^–1^ or absent for PA, which provided average values of 35.0 and 8.8 for the IA/PA ratios in R1 and R2, respectively (Table 3). Due to their high values, the IA/PA values for phase 3 were not included in the Figure 3C. According to the study by Ripley et al.,^51^ which is normally referenced in the literature for the discussion of the IA/PA ratio, the authors treated waste from chicken production in anaerobic reactors and indicated that the IA/PA values should be lower than 0.3. However, even with values higher than 0.3, it is possible to monitor the stability of anaerobic reactors that treat vinasse. For example, in UASB reactors in series, Barros et al.^5^ reported average IA/PA values of 0.86 and 0.63 for R1 and R2 reactors, respectively. The authors treated vinasse with an OLR of 45 g total COD (L d)^−1^ in R1 and observed an average volumetric methane production of 3.12 L CH_4_ (L d)^−1^ for the system.

The TVA values increased with increasing OLR, reaching concentrations close to 6000 mg L^–1^ in the effluents of the UASB reactors (Figure 2D). These values are the same as those reported by Barros et al.,^5^ of up to 6000 mg L^–1^ for operating UASB reactors in series and treated vinasse supplemented with filter cake with an OLR of 45 g total COD (L d)^−1^.

During anaerobic digestion, in the acidogenesis stage, large amounts of total volatile acids (TVAs) are produced.^52^ Anaerobic co-digestion of vinasse and molasses has high potential for TVA production. Sugarcane vinasse is a substrate that has high potential for TVA production because it contains a high biodegradable organic load,^53^ and molasses compounds such as sucrose (30 to 40%), fructose (5 to 12%), and glucose (4 to 9%),^15^ which are also easily degraded and converted to TVA.

The TVA values of the effluents of the UASB, R1 and R2 reactors, in phases 1, 2, and 4, were lower than those observed in the influent, indicating that their consumption occurred (Figure 2D) by acetogenic and methanogenic microorganisms. Supplementing the influent with filter cake contributed to nutritional balance, since the availability of nutrients is an important factor in anaerobic treatment because of the high concentrations of organic material in the vinasse and molasses.

In phase 3, because of the load shock, there was an increase in TVA in reactors R1 and R2, even with the removal of recirculation and the correction of the pH of the influent with lime milk during several periods. These changes indicated the predominant activity of acidogenic microorganisms in the anaerobic co-digestion of vinasse and molasses. Acidogenic microorganisms are responsible for the production of organic acids from the degradation of biodegradable organic matter, which results in an increase in the level of TVA in the reactor effluents.

The OLR reduction strategy in phase 4 played a crucial role in improving reactor system stability in terms of TVA, pH, and alkalinity (Figure 2A–D). This highlights the complexity and need for multifaceted approaches in the management of a series of reactors, emphasizing that operational optimization can be as effective as direct chemical remediation in achieving desirable results in wastewater treatment.

When the TVA parameters were analyzed together with TA, the average values of the TVA/TA ratio were 0.16 and 0.13, and 0.15 and 0.11 in R1 and R2, in phases 1 and 2, respectively. In phases 3 and 4, they were 2.83 and 1.82 and 0.52 and 0.44, respectively, in reactors R1 and R2 (Table 3). Values greater than 0.8 may indicate the inhibition of methanogenic archaea.^54^ In this experiment, with TVA/TA ratios above 0.8 during phase 3, occurred a reduction in volumetric methane production (VMP) (Figure 3). The stability of the TVA/TA ratio is more significant than the values of this parameter.^55^

### 3.2 Chemical Oxygen Demand, Suspended Solids (SS), and Biogas Production

The average values of total COD, dissolved COD, TSS, and VSS decreased from the influent to the effluents of the UASB reactor, R1 and R2, in phases 1, 2, 3, and 4 (Table 4).

**Table 4:** Average Values of Total Chemical Oxygen Demand (Total COD), Dissolved Chemical Oxygen Demand (Diss COD), Total Suspended Solids (TSS), Volatile Suspended Solids (VSS), and the VSS/TSS Ratio in the Influent and Effluents of the Upflow Anaerobic Sludge Blanket Reactors (UASB), in series (R1 and R2), Used in the Co-Digestion of Vinasse and Molasses Supplemented with Filter Cake, in Phases 1, 2, 3, and 4^a^

			Phases									
Parameter	Sample		1		2		3		4		VC*	F
Total COD	influent	average	4857	c	29,505	a	31,696	a	10,932	b	34	**
		VC	66		39		20		25			
	R1	average	2297	c	8125	b	21,701	a	7490	b	41	**
		VC	78		63		25		39			
	R2	average	1573	c	5245	b	16,701	a	5987	c	40	**
		VC	71		65		26		40			
Diss COD	influent	average	2408	d	15,098	b	24,965	a	7588	c	38	**
		VC	55		50		22		15			
	R1	average	998	c	4268	b	12,522	a	4344	b	44	**
		VC	71		79		33		29			
	R2	average	796	c	2464	b	10,108	a	3502	b	37	**
		VC	74		59		35		29			
TSS	influent	average	2203	ab	4694	a	3290	ac	1183	c	53	**
		VC	79		41		45		43			
	R1	average	2024	a	3089	a	3469	a	2250	a	98	**
		VC	99		40		50		48			
	R2	average	1305	b	2808	a	2296	ab	1294	b	55	**
		VC	78		47		43		82			
VSS	influent	average	1634	ac	3433	a	2334	ab	950	c	55	**
		VC	83		41		50		48			
	R1	average	1537	a	2313	a	2783	a	1784	a	68	**
		VC	99		47		59		51			
	R2	average	984	c	2048	b	1774	ab	1074	c	54	**
		VC	83		49		43		66			
VSS/TSS	influent	average	0.74	a	0.73	a	0.69	a	0.80	a	35	**
		VC	53		25		24		11			
	R1	average	0.76	a	0.73	a	0.79	a	0.78	a	21	**
		VC	26		20		17		14			
	R2	average	0.74	b	0.74	b	0.78	ab	0.83	a	25	**
		VC	33		17		14		36			

a Units: mg L ^–1^, except for VSS/TSS; VC: variation coefficient (%) of each phase. Different lowercase letters in the same line differ according to Tukey’s test at 5%. F - F test; **—Significant at 1% probability (*p* < 0.01); ns—not significant (*p* > 0.05); *p*—probability. VC*—variation coefficient of each parameters for all phases.

The average removal rates of total COD were 53, 30, and 67%, and 74, 31, and 83% in R1, R2 and R1 + R2, in phases 1 and 2, respectively (Figure 3A). The highest values of total COD removal were observed in phase 2, which may have occurred mainly because of the adaptation of the sludge to the substrate. In phases 3 and 4, total COD removal was lower than that in the previous phases, with values of 31, 23, and 47% and 33, 20, and 46% in R1, R2 and R1 + R2, in phases 3 and 4, respectively (Figure 3A).

This significant reduction in removal rates is directly related to the occurrence of acidification during phase 3, which negatively affects the entire system. The efficiency of total COD removal in the anaerobic treatment of vinasse also decreased as the OLR increased.^56^

Phase 3 was particularly challenging, compromising the efficiency of anaerobic treatment. Acidification may have contributed to a decrease in the capacity of microorganisms to degrade organic matter, resulting in lower total COD removal values. These adverse conditions affected both R1 and R2 and, consequently, the combined efficiency of the system.

However, after recovery in phase 4, the total COD removal rates remained stable, indicating that the adopted strategy was effective at reestablishing the system equilibrium.

The VSS/TSS ratio decreased from the influent to the effluents of R1 (Table 4) in phase 4 , indicating that there was an increase in fixed suspended solids because of the consumption of volatile solids. The average values of removal efficiencies of TSS and VSS in the system composed of the UASB reactors, R1 + R2, were 41 and 40%; 39 and 40%, respectively, in phases 1 and 2.

The dissCOD/totalCOD ratios were 0.49, 0.43, and 0.50; 0.51, 0.52, and 0.47; 0.78, 0.57, and 0.60; and 0.69, 0.58, and 0.58 in the influent and effluents of R1 and R2, in phases 1, 2, 3, and 4, respectively. These values are lower than those observed, for example, by Barros^57^ (0.84 to 0.86), in the influent of UASB reactors treated with vinasse, with an OLR of up to 11.5 g total COD (L d)^−1^. The lowest values of dissCOD/totalCOD observed in this work, mainly in phases 1 and 2, may have occurred because the co-digestion of vinasse with molasses supplemented with filter cake contributed to the conversion of soluble compounds (dissCOD) to acids and subsequently to biogas, indicated by the average removals of diss COD of 66 and 82%, respectively, for reactors R1 + R2 in phases 1 and 2 (Figure 3A).

Increased volumetric methane production (VMP) were observed in the UASB reactors, R1 and R2, with increasing OLR, which reached values of up to 5.794 and 1.288 L CH_4_ (L d)^−1^, respectively, in phase 2 (Figure 3B).

The average values of VMP of reactors R1 and R1 + R2 in phases 1, 2, 3, and 4 were 0.52, 3.39, 0.73, and 1.14 L CH_4_ (L d)^−1^ and 0.39, 2.55, 0.67, and 1.00 L CH_4_ (L d)^−1^, respectively (Tables 5 and 6), with recirculation of the effluent from R2 taking advantage of the alkalinity in phases 1, 2, and 4.

**Table 5:** Average Values of Volumetric Methane Production (VMP), and Specific Methane Production (SMP), in the Upflow Anaerobic Sludge Blanket Reactor (UASB), in series (R1 and R2), Used in the Co-Digestion of Vinasse and Molasses Supplemented with Filter Cake, in Phases 1, 2, 3, and 4^a^ and Potential of Electrical Energy Production from Biogas (En)

			Phases									
Parameter	Sample		1		2		3		4		VC*	F
VMP	R1	average	0.52	d	3.39	a	0.73	c	1.14	b	52	**
		VC	70		39		31		34			
	R2	average	0.14	c	0.71	a	0.54	b	0.70	a	61	**
		VC	103		40		79		21			
	R1 + R2	average	0.39	d	2.55	a	0.67	c	1.00	b	48	**
		VC	71		38		30		28			
SMP	R1	average	0.21	b	0.17	c	0.09	d	0.35	a	49	**
		VC	56		32		52		37			
	R2	average	0.11	c	0.20	b	0.06	d	0.30	a	90	**
		VC	122		68		70		73			
	R1 + R2	average	0.19	b	0.16	b	0.08	c	0.33	a	49	**
		VC	66		28		42		37			
En	R1	average	0.022	b	0.147	a	0.032	b	0.051	b	52	**
		VC	70		40		31		34			
	R2	average	0.003	b	0.014	ab	0.011	ab	0.014	b	61	**
		VC	103		40		79		20			
	R1 + R2	average	0.029	b	0.162	a	0.043	b	0.065	b	48	**
		VC	71		39		30		28			

a VC: variation coefficient (%). VMP: L CH_4_ (L d)^−1^; SMP: L CH_4_ (g total COD removed)^−1^; Different lowercase letters in the same line differ according to Tukey’s test at 5%. F-F test. **—Significant at 1% probability (p < 0.01); ns—not significant (*p* > 0.05); *p*—probability. VC*—variation coefficient of each parameters for all phases.

**Table 6:** Results of the Anaerobic Co-Digestion of Vinasse and Sugarcane Molasses^a^

Reactor (volume)	Influent	*T* (°C)	OLR (g total COD (L d)^−1^)	total COD removed (%)	VMP (L CH_4_ (L reactor d)^−1^)	SMP (L CH_4_ g^–1^ total COD removed)	Alkali and nutrients	References
AnSBBR (6 L)	molasse (33%)+ vinasse (67%)^b^	25	6.9–13.7	1–20	0–13.5^d^	-	-	^60^
AFBR (T) (1.9 L) + AFBR (M) (1.9 L)	molasse (50%) + vinasse (50%)^b^	55 (T), 30 (M)	6–27	58.4–84.5 (T), 47.5–84.5 (M)	1.2–3.8 (T), 1.2–3.4 (M)	0.128–0.274 (T), 0.145–0.307 (M)	NaOH and HCl solutions, with NaHCO_3_ added at a ratio of 0.3–0.5 g NaHCO_3_ g^–1^ COD	^21^
Batch biodigesters (310 mL)	molasse (75; 50; 25) + vinasse (25; 50; 75)^c^	35	-	2.9–40	0.009–0.013	-	NaHCO_3_	^17^
UASB (9.75 L)	molasse (50%) + glucose (50%)^b^	35	1.5–18	52–88	0.08–4.3	0.23–0.35	NaHCO_3_; CH_4_N_2_O; KH_2_PO_4_	^20^
UASB – R1 (12.1 L) + UASB – R2 (5.6 L)	molasse (50%) + vinasse (50%)^b^	35	4.8–29.5 (R1), 5.0–17.6 (R2)	53–74 (R1), 30–31 (R2); 67–83 (R1 + R2)	0.52–3.39 (R1), 0.14–0.71 (R2); 0.39–2.55 (R1 + R2)	0.21–0.17 (R1), 0.11–0.20 (R2); 0.19–0.16 (R1 + R2)	filter cake; recirculation of effluent from R2	this study (phases 1 and 2)
UASB – R1 (12.1 L) + UASB – R2 (5.6 L)	molasse (50%) + vinasse (50%)^b^	35	31.7 (R1); 47.2 (R2)	31 (R1); 23 (R2); 47 (R1 + R2)	0.73 (R1); 0.54 (R2); 0.67 (R1 + R2)	0.09 (R1); 0.06 (R2); 0.08 (R1 + R2)	filter cake; calcium hydroxide was used as an alkalizing agent	this study (phase 3)
UASB – R1 (12.1 L) + UASB – R2 (5.6 L)	molasse (50%) + vinasse (50%)^b^	35	11.0 (R1); 16.2 (R2)	33 (R1); 20 (R2); 46 (R1 + R2)	1.14 (R1); 0.70 (R2); 1.00 (R1 + R2)	0.35 (R1); 0.30 (R2); 0.33 (R1 + R2)	filter cake; recirculation of effluent from R2	this study (phase 4)

a AnSBBR: anaerobic reactor operated in sequence batch and/or fed batch with immobilized biomass on inert support and mechanical agitation; AFBR (M): mesophilic anaerobic fluidized bed reactor; AFBR (T): thermophilic anaerobic fluidized bed reactor ; UASB – upflow anaerobic sludge blanket reactor; totalCOD: total chemical oxygen demand; OLR: organic loading rate; VMP: volumetric methane production; SMP: specific methane production; T: temperature;^b^ in terms of COD; ^c^ in terms of volume (%); ^d^ (mol H_2_ L^–1^ d^–1^).

For example, the average VMP obtained was 0.94 L CH_4_ (L d)^−1^ in a UASB reactor treated vinasse with an OLR of 11.5 g total COD (L d)^−1^ using effluent recirculation in the mesophilic temperature range.^57^ Barros et al.^5^ obtained VMP values of 2.1 and 1.2 L CH_4_ (L d)^−1^ in the UASB, R1 and R2 reactors, respectively, with the application of an OLR of 33 g total COD (L d)^−1^ in R1, treating vinasse supplemented with filter cake in the thermophilic temperature range, with the recirculation of the effluent from R2 to take advantage of alkalinity. VMP values of 10.24 L CH_4_ (L d)^−1^ were reported by Barbosa et al.,^19^ who treated vinasse in a UASB reactor with an OLR of 33 g of total COD (L d)^−1^ and with the use of sodium bicarbonate to maintain the alkalinity of the influent. However, despite the additional stability of anaerobic reactors for treating vinasse, the use of high doses of chemicals negatively affects the economic favorability of scaling up anaerobic digestion plants.^58^

The average values of specific methane production (SMP) were 0.21, 0.17, 0.09, and 0.35 L CH_4_ (g total COD removed)^−1^ for R1 and 0.19, 0.16, 0.08, and 0.33 L CH_4_ (g total COD removed)^−1^ for R1 + R2 in phases 1, 2, 3, and 4, respectively. The theoretical value is 0.35 L CH_4_ (g total COD removed)^−1^.^59^ This finding indicates that for the system (R1 + R2), approximately 54, 48, and 100% of the theoretical methane yield was obtained in phases 1, 2, and 4, respectively.

The estimated values for potential of electrical energy production from biogas (En) were 0.029, 0.162, 0.043, and 0.065 kWh d^–1^ (Table 5) for the conditions studied, in phases 1, 2, 3, and 4, respectively. Considering the daily influent vinasse flow rate, for phase 2, when the highest volumetric methane production occurred in the UASB reactors, 19 kWh d^–1^ can be produced per m^3^ of vinasse; that is, 68% of the electrical energy required to process one ton of sugarcane.

The UASB reactor, R2, contributed approximately 10, 9, 25, and 21% of the En estimate in phases 1, 2, 3, and 4, respectively. This highlights the importance of R2, especially in situations with shock loads, as observed in phase 3. For the conditions of phases 1 and 2, the economic evaluation of installing UASB reactors, in series, for the co-digestion of vinasse and molasses supplemented with filter cake is suggested, considering the En produced in R1 and R2.

Analysis of the percentages of methane in the biogas in the R1 and R2 reactors revealed significant variations throughout different phases of the study. In R1, the average values were 70.4% (phase 1), 71.5% (phase 2), 72.6% (phase 3), and 73.4% (phase 4). In R2, more pronounced changes were observed: 60.6% (phase 1) and 39.5% (phase 2), indicating variations in the efficiency of methane production according to the operational conditions and the influence of added substrates, such as molasses. In comparison, in a study with vinasse, Barros et al.^57^ reported decreases in the percentage of methane in the biogas in the R1 and R2 reactors with increasing OLR, reaching minimum values of 2% and 12%, respectively.

The VMP and SMP values obtained in this work and in similar studies described in the literature are listed in Table 6. The different compositions of the influent and types of anaerobic reactors, in addition to the operational parameters, such as HDT, temperature, and COD, make comparisons of VMPs and SMPs difficult. However, the average VMP results obtained in phase 2 were 2.55 L CH_4_ (L d)^−1^ for R1 + R2, even without the addition of an alkalizing agent.

### 3.3 Nutrients

#### 3.3.1 N and P

Microbial activity is closely related to the presence of ammoniacal nitrogen (Am-N), which serves as a nitrogen source for microorganisms involved in anaerobic digestion.^61^ Am-N values of up to 160 mg L^–1^ were observed. The Kjeldahl nitrogen (KN) values decreased from the influent to the effluent of the UASB reactors, R1 and R2 (Table 7), for which the average removal rates did not significantly differ (12, 17, 17, and 13%, respectively) in R1 + R2, in phases 1, 2, 3, and 4, respectively.

**Table 7:** Average Concentrations of Kjeldahl Nitrogen (KN), Total Phosphorus (Total-P), and Ammoniacal Nitrogen (Am- N), and the Ratio of Total COD/KN/Total-P in the Influent and Effluents, and the Removal of KN and Total-P (in %) in the Upflow Anaerobic Sludge Blanket Reactors (UASB), in series (R1 and R2), Used in the Co-Digestion of Vinasse and Molasses Supplemented with Filter Cake, in Phases 1, 2, 3, and 4^a^

			Phases									
Parameter	Sample		1		2		3		4		VC*	F
Am-N	influent	Average	18	c	58	b	68	b	123	a	57	**
		VC	72		66		61		27			
	R1	Average	22	b	41	b	41	b	145	a	61	**
		VC	77		64		94		24			
	R2	Average	27	b	45	b	44	b	160	a	52	**
		VC	68		65		92		29			
KN	influent	Average	136	b	430	a	405	a	342	a	53	**
		VC	75		54		48		10			
	R1	Average	123	b	273	a	337	a	301	a	43	**
		VC	71		28		46		8			
	R2	Average	114	b	232	a	273	a	261	a	42	**
		VC	69		24		46		7			
	removal (R1 + R2)	Average	12	a	17	a	17	a	13	a	96	n.s.
		VC	100		68		94		42			
Total-P	influent	Average	21	d	75	b	61	c	122	a	46	**
		VC	39		29		65		40			
	R1	Average	22	c	53	a	40	b	58	a	40	**
		VC	62		32		39		13			
	R2	Average	20	c	32	ac	29	ab	47	a	47	**
		VC	52		60		43		14			
	removal (R1 + R2)	Average	-	-	63	a	48	a	57	a	54	**
		VC	-		42		40		24			
totalCOD/KN/total-P	influent	Average	350:10:1.5		350:5:0.9		350:4:0.7		350:11:3.9			
	R1	Average	350:18:3.3		350:13:3.2		350:5:0.6		350:14:2.7			
	R2	Average	350:25:4.5		350:18:3.3		350:6:0.6		350:12:2.8			

a Units: mg L ^–1^, except removal (%). VC: variation coefficient (%) of each phase. total COD: total chemical oxygen demand. Different lowercase letters in the same line differ according to Tukey’s test at 5%. F - F test. **—Significant at 1% probability (*p* < 0.01); ns—not significant (*p* > 0.05); *p*—probability. VC*—variation coefficient of each parameters for all the phases.

For total-P, no removals were observed in phase 1, and average values of efficiency of 63, 48, and 57% were observed in phases 2, 3, and 4, respectively, for R1 + R2 (Table 7). In the effluent of R2, average concentrations of KN and total-P of 114, 232, 273, and 261 mg L^–1^ and 20, 32, 29, and 47 mg L^–1^ were also observed in phases 1, 2, 3, and 4, respectively. This is an important characteristic, since the excess effluent could be used in the fertigation of sugarcane, for example.

The use of filter cake in the co-digestion of vinasse and molasses aims to meet the COD, KN, and total-P ratios and ensure an adequate nutritional balance for the microorganisms involved in anaerobic digestion. The COD/KN/total-P ratio in the effluent of R1 and R2 was higher than the recommended 350:5:1 for anaerobic digestion^23^ in phases 1, 2, and 4, which may have contributed to the high VMP (Figure 3B). In phase 3, phosphorus deficiency was observed, which may have occurred because of its precipitation with calcium from calcium hydroxide used to correct the pH. In anaerobic reactors, phosphorus removal can occur because of the formation of hydroxyapatite (Ca_5_(PO_4_)_3_(OH)).^49^

The filter cake used in this study presented important characteristics that may influence anaerobic digestion. With a pH of 6.2, the cake has an organic carbon content of 341 g kg^–1^, which makes it a rich source of organic matter. In addition, it contains 17 g kg^–1^ of total nitrogen, providing essential nutrients for microbial growth. A calcium concentration of 301.4 g kg^–1^, a magnesium concentration of 81.2 g kg^–1^, and a potassium concentration of 20 g kg^–1^ contributed to the nutritional balance of the system, whereas a total phosphorus concentration of 16 g kg^–1^ and a sulfur concentration of 17 g kg^–1^ were essential for metabolic processes. Trace metals also stand out, and the copper, iron, manganese, and zinc, which play crucial roles in enzymatic activity and methanogenesis efficiency, increasing the productivity of the UASB system by increasing the degradation of organic matter and the conversion of substrates into biogas.^62^

This acidic profile of vinasse, although challenging for anaerobic digestion, is rich in organic matter that can be used for biogas production. The combination of vinasse, molasse, and filter cake, which has a pH of 6.2 and high contents of carbon, nitrogen, and minerals, can help balance the system conditions, thus promoting microbial activity. pH correction and the addition of essential nutrients from the filter cake can mitigate the inhibitory effects of vinasse acidity, promoting a more favorable environment for COD degradation and increasing the efficiency of methane production in UASB reactors.^63^

#### 3.3.2 Sulfide and Sulfate

The main source of sulfate in vinasse is sulfuric acid, which is used in distilleries to correct the pH of the must before ethanol fermentation. In addition, in molasses, the source of sulfate is sulfur dioxide, which is used in industries to produce white sugar. According to the literature, the sulfate content in vinasse varies from 500 to 1500 mg L^–1^.^64^

In the influent of the UASB reactors, increasing concentrations of sulfate and sulfide ions were observed with increasing OLR, reaching values of approximately 1000 and 10 mg L^–1^, respectively (Figure S2). In phase 3, there was a significant increase in the concentrations of sulfate ions in the influent and effluents. This significant increase can be attributed to acidification and the possible increase in the availability of sulfur in the substrates used. During acidification, sulfate may be released from sulfur-containing organic compounds, contributing to the observed increase.

In phase 4, sulfate concentrations decreased to approximately 500 mg L^–1^ in the influent but remained lower in the effluents. This may be the result of the system recovery after OLR reduction. By reducing the amount of available organic matter, sulfate production from the decomposition of sulfur-containing organic compounds was reduced.

Sulfate removal was observed in the UASB reactors, R1 and R2, which is important since high sulfate concentrations do not cause toxicity to methanogenic archaea. However, increasing the proliferation of sulfate-reducing bacteria (SRB) can cause a decrease in methane production. This is because SRB compete with methanogenic archaea for substrates, as they have a greater affinity for hydrogen and acetate and support a greater pH range.^47^

Dissolved sulfide concentrations of 100 to 800 mg L^–1^ can inhibit anaerobic microorganisms;^65^ however, the values observed in this work were lower than 10 mg L^–1^, indicating that no sulfide toxicity occurred.

#### 3.3.3 K, Na, Ca, Mg, Fe, Mn, Cu, Zn, Co, and Ni

Metals are essential components of cofactors and enzymes^62^ ^,^ and the addition of filter cake can be an important alternative for supplementing nutrients in the influent of UASB reactors, co-digesting vinasse and molasses.

In this work, the highest average VMP, 2.55 L CH_4_ (L d)^−1^, occurred for the R1 + R2 system in phase 2, with an OLR of 29.5 g COD_total_ (L d)^−1^ in R1. In this phase, the average concentrations of K, Ca, Mg, Fe, Mn, Cu, Zn, Co, and Ni in the influent were 1286.3, 6.65, 178.64, 10.75, 2.57, 0.24, 0.50, 0.13, and 0.18 mg L^–1^, respectively (Tables S1–S3).

In the study carried out by Barros et al.,^5^ thermophilic UASB reactors were used in series, when they treated vinasse supplemented with filter cake, and the highest average VMP of 3.12 L CH_4_ (L d)^−1^ for the R1 + R2 system was observed with the application of an OLR of 45 g total COD (L d)^−1^ in R1. The authors reported higher average concentrations of Mg, Fe, Mn, Zn, and Cu in the influent (15.4, 25.5, 5.2, 10.4, and 3.8 mg L^–1^, respectively) and they attributed the good performance of the UASB reactors to the higher values of mainly Fe, which increased the biomass concentrations of bacteria and methanogenic archaea involved in propionate degradation.

Decreases were observed in the average concentrations of Ca, Fe, Na, Mn, Cu, and Zn from the influent to the effluents of the UASB reactors R1 and R2. However, even as R2 decreased, it remained in the effluent. This is important, mainly because biodigested effluent can be used for the fertigation of sugarcane fields. Potassium, for example, as reviewed by Parsaee et al.^66^ ^,^ does not decrease after the biodigestion of vinasse and can be used as fertilizer, as also observed in this work.

Trace metal deficiency can cause problems in the performance of anaerobic systems in terms of VMP and increased acid concentration.^67^ The ratios of Fe, Ni, Co, and Zn for the anaerobic digestion of glucose in the mesophilic temperature range should be 0.20, 0.0063, 0.017, and 0.049 mg g^–1^ COD removed, respectively.^68^

In this work, the values of ratio concentration per total COD removed were observed in R1: for Fe, 0.0537, 0.1614, 0.3055, and 0.0723 mg g^–1^ COD removed were obtained in phases 1, 2, 3, and 4, respectively. For Co, the values were 0.00029, 0.00197, 0.00177, and 0.00279 mg g^–1^ total COD removed in phases 1, 2, 3, and 4, respectively. For Zn, the concentrations were 0.00191, 0.00758, 0.00367, and 0.00279 mg g^–1^ total COD removed in phases 1, 2, 3, and 4, respectively. For Ni, 0.00015, 0.00273, 0.00544, and 0.00558 mg g^–1^ total COD were removed in phases 1, 2, 3, and 4, respectively.

### 3.4 Total and Volatile Solids of Sludge from the UASB reactors, in series (R1 and R2)

The highest values of total solids (TS) and volatile solids (VS) in the sludge were observed in R1 (Figure S1), although reactors R1 and R2 received 30% of their volumes with inoculum. This may have occurred because of the higher OLR applied in R1 and added to the methanogenic activity, which led to an increase in the multiplication of microorganisms and, consequently, an increase in VMP.

The VS/TS ratio of the inoculated sludge was 0.57. The presence of high ratios of VS in relation to TS indicates the predominance of organic matter in the sludge of reactors R1 and R2, and this was observed in phases 1, 3, and 4, with VS/TS ratios above 0.6 (Figure S1). This higher ratio suggests the presence of microorganisms, which play a fundamental role in the degradation of the organic compounds present. This observation is consistent with the elevated conversion rates of chemical oxygen demand (COD) to methane observed in reactors R1 and R2, mainly in phases 1, 2, and 4.

During phase 3, with shock load, organic acids increased, and the pH decreased, which may influence the adhesion and cohesion of the biomass to the reactor bed. In addition, the presence of organic acids may create a more favorable environment for the growth of certain acidogenic microorganisms, leading to an apparent increase in the biomass in the reactor.

However, as the recovery phase is set in and corrective measures or operational strategies are implemented, such as reducing the organic load or stabilizing the pH, the system conditions improve. As equilibrium is restored, a reduction in the overgrowth of these microorganisms adapted to acidified conditions may occur, leading to an apparent decrease in the sludge in the reactors.

### 3.5 Microbial Diversity, Functional Prediction, and Metabolism Pathways in UASB Reactors

The evaluation of the microbial composition was performed on the inoculum sludge and on the sludge contained in the UASB reactors, in series (R1 and R2) , on days 348 and 505 during phases 3 and 4, respectively (Figure 4 and 5). The characterization of the microbial community profile was conducted at the genus taxonomic level using the last identifiable taxonomic level.

**Figure 4:**
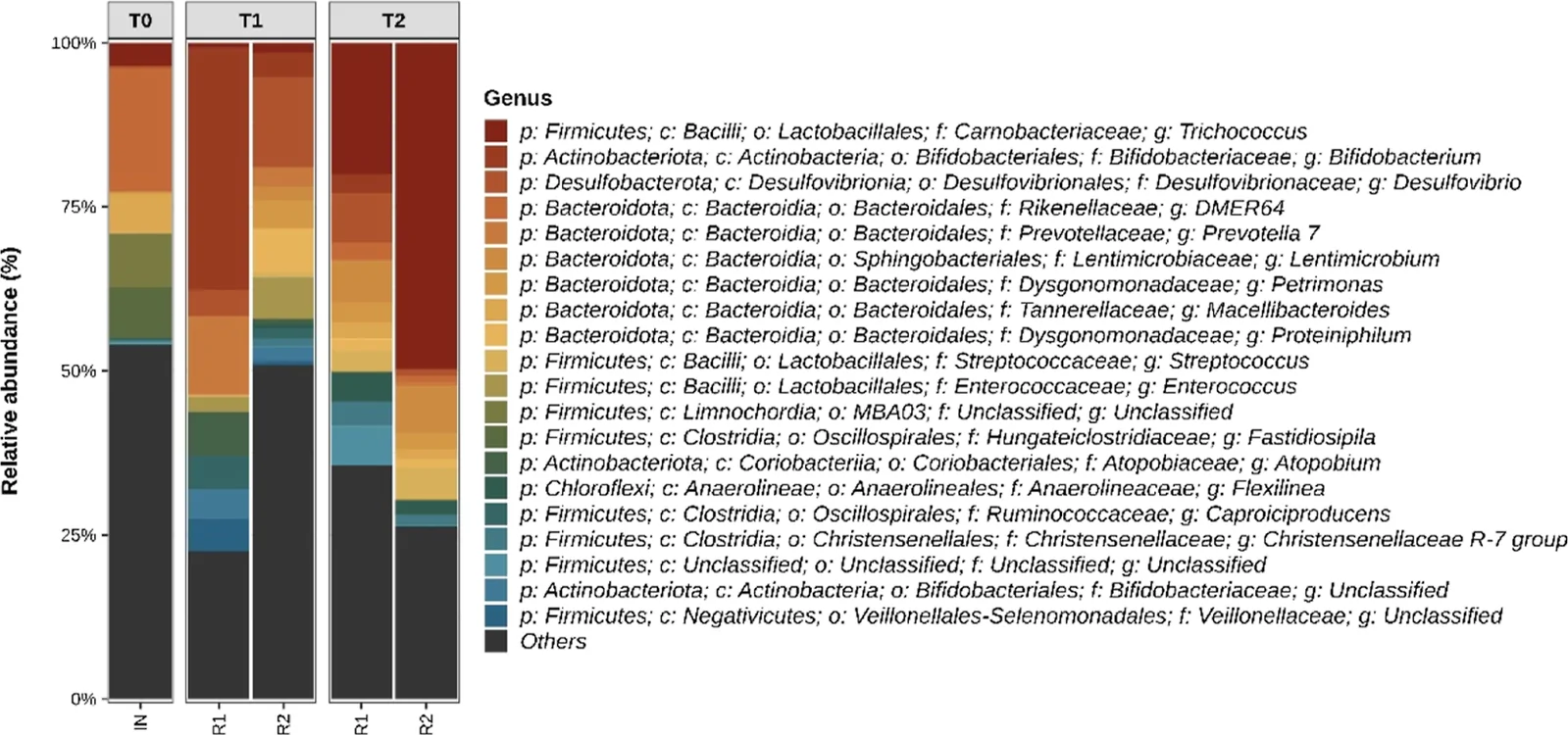
Taxonomy and relative abundance of genera in the Bacteria domain of the inoculum sludge (T0) and in the sludge from the UASB reactors, R1 and R2, in phases 3 (T1) and 4 (T2).

**Figure 5:**
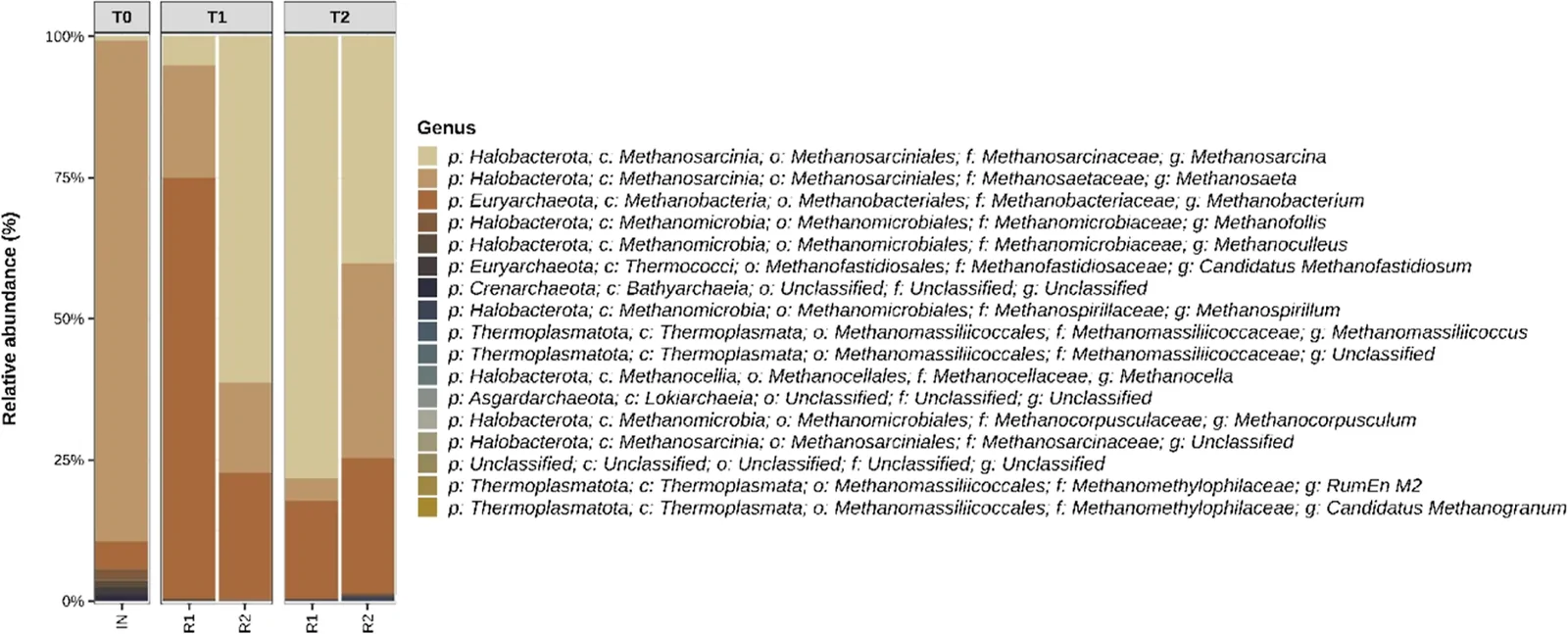
Taxonomy and relative abundance of the genera of the Archaea domain in the inoculum sludge (T0) and in the sludge from the UASB reactors, R1 and R2, in phases 3 (T1) and 4 (T2).

The most abundant bacterial phylum in the inoculum was Firmicutes, followed by Bacteroidota and Proteobacteria. In the UASB reactors, in series (R1 and R2), the phylum that presented the highest relative abundance was Firmicutes, followed by Bacteroidota and Actinobacteria, which are recognized as hydrolytic fermentative bacteria in the anaerobic digestion process.^69^ The bacterial phyla Firmicutes and Bacteroidetes play crucial roles in anaerobic digestion, especially in environments rich in organic matter such as UASB reactor sludges.

Bacteria of the phylum Firmicutes, for example, are capable of fermenting complex carbohydrates and producing short-chain fatty acids, which are important intermediates in anaerobic digestion.^70^ Furthermore, the metabolic diversity of bacteria is highlighted, allowing the degradation of a wide range of polysaccharides and phenolic compounds, with a positive effect on the efficiency of the biogas process.^71^

In R1, in phase 3, the observation of the Actinobacteria phylum, especially the *Bifidobacterium* genus, revealed important dynamics during anaerobic fermentation. Bacteria of this genus are known for their ability to perform heterofermentative fermentation, generating organic acids such as lactic acid and acetic acid.^72^ However, excessive acidification had adverse effects on the reactor, resulting in a significant decrease in pH, which can inhibit the activity of essential microorganisms, such as methanogenic archaea, compromising biogas production.^73^ Proper management of operating conditions is, therefore, crucial to maximizing the desired metabolic activity and ensuring system stability.^71^ Although less abundant, the phylum Actinobacteria also contributes to the degradation of recalcitrant compounds, complementing fermentative activity and the conversion of organic matter into biogas.^74^

In the sludge from R1 and R2, in the phase 4, the phylum Firmicutes and genus *Trichococcus* prevailed. *Trichococcus* plays an important role in fermentation and can produce various acids, such as lactic acid, formic acid, and acetic acid, from glucose.^75^ The production of formic acid, for example, can serve as an important intermediate, favoring the growth of methanogenic archaea that use these compounds for biogas generation.^76^ The metabolic diversity observed in *Trichococcus* suggests that this bacterium not only resists variations in reactor conditions but also plays an active role in the recovery and overall efficiency of the anaerobic digestion process. This adaptability is essential for the robustness of the system, allowing it to recover from periods of stress and maintain optimal levels of biogas production.

In the Archaea domain, the greatest abundance of the phylum Halobacterota and genus *Methanosaeta* was observed in the inoculum. In R1 of phase 3, *Methanobacterium* prevailed, which are hydrogenotrophic archaea that use CO_2_, H_2_, and formate.^77^ The ability of *Methanobacterium* to metabolize hydrogen and carbon dioxide is particularly relevant in UASB reactors, where these conditions can be favored. The interaction between different groups of archaea and bacteria in anaerobic reactors is essential for optimizing biogas production, and the presence of hydrogen can increase methanogenic activity.^77^ In R2 of phase 3 and R1 and R2 of phase 4, the genus *Methanosarcina* prevailed. *Methanosaeta* are known for their ability to efficiently convert acetate to methane, which is crucial for anaerobic digestion.^23^ In contrast, *Methanosarcina* is more versatile, utilizing both acetate and other substrates, including methanol, demonstrating metabolic flexibility that may contribute to the stability of the system under variable conditions.^5^

Despite the predominance of *Methanosaeta* in the inoculum, the versatility of *Methanosarcina* and the ability of *Methanobacterium* to utilize hydrogen and CO_2_ were essential for the metabolic diversity and stability of the anaerobic digestion system.^76^

The results of 16S rRNA sequencing were used for functional prediction using FAPROTAX to identify changes in the functional microorganisms in both R1 and R2 throughout different phases. According to the cycling function prediction by FAPROTAX (Figure 6), the dominant functions in R1 and R2, in phase 3, were chemoheterotrophy > fermentation > animal parasites or symbionts.

**Figure 6:**
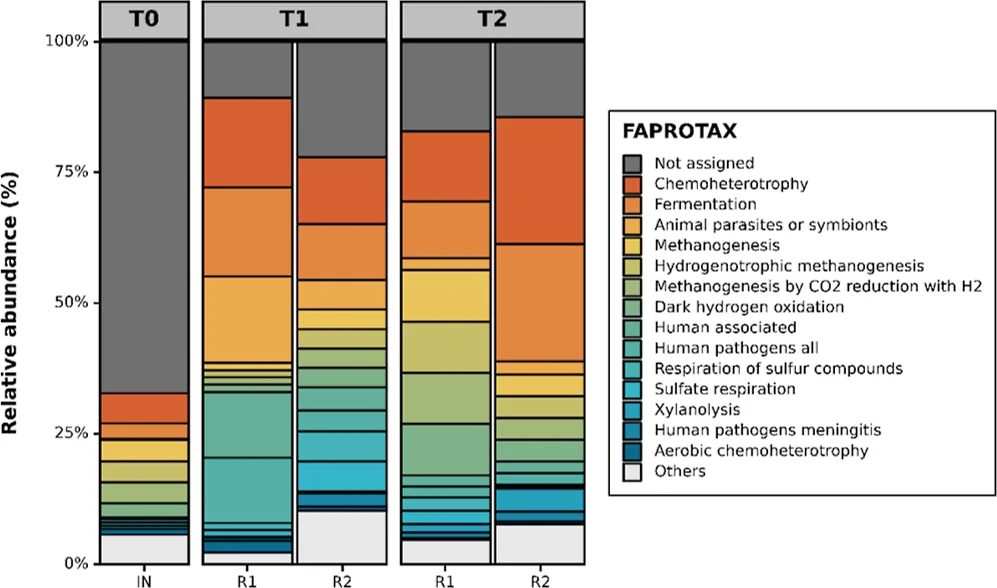
Functional annotation of prokaryotic taxa (FAPROTAX) analysis to predict metabolic processes in the inoculum sludge (T0) and in the sludge from the UASB reactors, in series (R1 and R2) in phases 3 (T1) and 4 (T2).

In R1, in phase 4, with the recovery of the shock load, an increase in functions such as methanogenesis, hydrogenotrophic methanogenesis, and methanogenesis by CO_2_ reduction with H_2_ was observed. In R2, from phase 4, the functions that prevailed were chemoheterotrophy and fermentation. In R1 and R2, the metabolism pathways (KEGG) of carbohydrates → amino acids → cofactors and vitamins → energy were observed (Figure 7).

**Figure 7:**
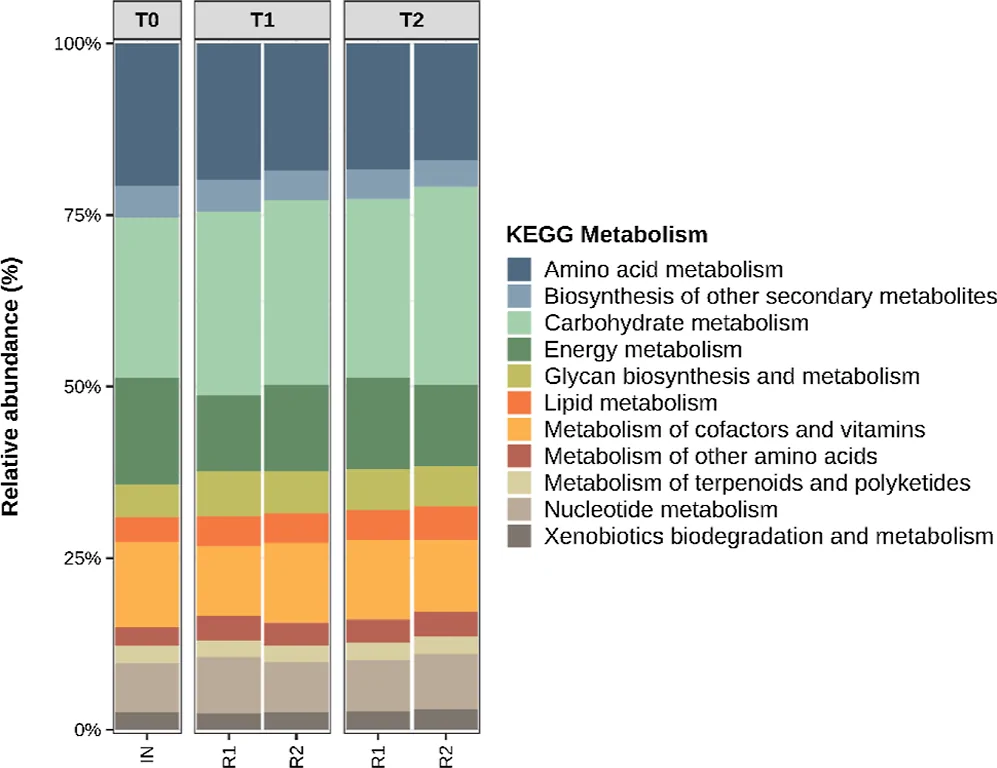
Kyoto Encyclopedia of Genes and Genomes (KEGG) metabolism in the inoculum sludge (T0) and in the sludge from the UASB reactors, in series (R1 and R2) , in phases 3 (T1) and 4 (T2).

## 4 Conclusion

Volumetric methane production of up to 4.3 L CH_4_ (L d)^−1^ was observed for R1 + R2, for an OLR applied in R1 of up to 45.0 g of total COD (L d)^−1^, during the anaerobic co-digestion process of vinasse and molasses supplemented with filter cake. This indicates the viability of the anaerobic process for the use of byproducts from the sugar-energy sector in the production of bioenergy. With increasing OLR, system collapse was observed with acidification of the reactors. The reduction in the organic load rate, the correction of the pH with calcium hydroxide, and the recirculation of the effluent from R2 were crucial for the reestablishment of the balance, which highlights the robustness of the anaerobic systems. With decreasing OLR after shock loading, the abundances of the genera *Trichococcus* and *Methanosarcina* increased over those of *Bifidobacterium* and *Methanobacterium* in the sludge of the UASB reactor (R1).

## Supplementary Material



## Notes

Average values of K, Na, Ca, Mg, Fe, Mn, Cu, Zn, Ni, and Co in the influent and effluents of the upflow anaerobic sludge blanket reactors (UASB), in series (R1 and R2), for the co-digestion of vinasse and molasses supplemented with filter cake, in phases 1, 2, 3, and 4; Total solids (TS) and volatile solids (VS) at the sludge collection points of the upflow anaerobic sludge reactors (UASB) in series (R1 and R2) during the co-digestion of vinasse with molasses supplemented with filter cake in phases 1, 2, 3, and 4; P1: base of the reactor; Values of sulfate and sulfide ions in the influent and effluents of the upflow anaerobic sludge blanket reactors (UASB), in series (R1 and R2), in the co-digestion of vinasse with molasses supplemented with filter cake, in phases 1, 2, 3, and 4 (PDF)
