# Supplementary material for: Co-Digestion of Sugarcane Biorefinery Byproducts in Upflow Anaerobic Sludge Blanket Reactors, in Series, with Application of High Organic Loading Rate
Source: ACS Omega. 2026 Aug 21;11(35):52372–88. doi: 10.1021/acsomega.5c06244 (PMC13564006; doi:10.1021/acsomega.5c06244)
Supplement: Supplementary file 1 [file ao5c06244_si_001.pdf]

Supporting Information

CO-DIGESTION OF SUGARCANE BIOREFINERY BYPRODUCTS IN UPFLOW ANAEROBIC SLUDGE BLANKET (UASB) REACTORS, IN SERIES, WITH APPLICATION OF HIGH ORGANIC LOADING RATE

*Bruna R. S. Sturaro; Lucas A. L. de Carvalho; Daniel G. Pinheiro; Roberto A. de Oliveira; Rose M. Duda**

* Graduate Program in Agricultural and Livestock Microbiology, São Paulo State University (UNESP), School of Agricultural and Veterinary Sciences, Jaboticabal, SP, 14884-900, Brazil and Technology Faculty "Nilo de Stéfani", Paula Souza Center, Jaboticabal, SP, Brazil. Av. Eduardo Carlos Zambianchi, n. 31, Vila Industrial, 14883-130, Jaboticabal, SP, Brazil. E-mail: rose.duda@unesp.br

Table S1. Average values of K, Na, Ca and Mg in the influent and effluents of the upflow anaerobic sludge blanket reactors (UASB), in series (R1 and R2) for the co-digestion of vinasse and molasses supplemented with filter cake, in phases 1, 2, 3 and 4.

Parameter	Samples		Phases									
			1		2		3		4		VC*	F
K	Influent	Average	23.6	c	1286.3	ab	2637.5	a	1672.7	a	75	**
		VC	78		114		40		16			
	R1	Average	22.2	c	1161.0	ab	2449.3	a	1776.7	ac	66	**
		VC	69		109		33		15			
	R2	Average	22.6	c	1157.1	ab	2508.1	a	1688.3	ab	67	**
		VC	59		109		32		21			
Na	Influent	Average	15.2	a	79.7	a	207.7	a	70.5	a	159	n.s.
		VC	41		105		131		28			
	R1	Average	14.4	b	66.6	ab	184.6	a	67.7	ab	133	**
		VC	28		86		109		27			
	R2	Average	15.0	b	70.2	ab	150.7	a	66.9	ab	96	**
		VC	18		91		78		44			
Ca	Influent	Average	5.2	b	6.6	b	291.9	a	234.0	ab	146	**
		VC	56		54		102		57			
	R1	Average	5.2	b	5.3	b	276.4	a	261.1	a	120	**
		VC	44		48		82		71			
	R2	Average	4.9	b	5.4	b	280.0	a	233.2	a	122	**
		VC	36		51		83		70			
Mg	Influent	Average	2.9	b	178.6	ab	238.0	ab	300.9	a	96	**
		VC	63		115		44		31			
	R1	Average	2.8	b	152.2	ab	204.5	ab	294.4	a	111	**
		VC	52		114		38		29			
	R2	Average	2.9	b	138.4	ab	290.0	a	312.3	a	94	**
		VC	42		130		52		41			

Units: mg L^{-1}. VC: variation coefficient in % of each phase. Different lowercase letters in the same line differ according to Tukey's test at 5%.F- F test. ** - Significant at 1% probability ($p<0.01$); ns - not significant ($p>0.05$); p-probability. VC*: coefficient of variation of each parameter for all phases.

Table S2. Average values of Fe, Mn, Cu and Zn in the influent and effluents of the upflow anaerobic sludge blanket reactors (UASB), in series (R1 and R2) for the co-digestion of vinasse and molasses supplemented with filter cake, in phases 1, 2, 3 and 4.

Parameter	Samples		Phases									
			1		2		3		4		VC*	F
Fe	Influent	Average	3.6	b	10.7	ab	24.1	a	6.2	b	86	**
		VC	103		63		72		76			
	R1	Average	2.7	b	9.7	ab	27.2	a	6.0	b	106	**
		VC	84		117		76		30			
	R2	Average	1.8	b	9.3	ab	23.7	a	5.4	b	100	**
		VC	83		84		77		23			
Mn	Influent	Average	1.3	ab	2.6	a	0.23	b	0.05	b	79	**
		VC	78		55		178		4			
	R1	Average	1.2	bc	1.7	a	0.20	b	0.05	c	78	**
		VC	56		63		170		9			
	R2	Average	1.0	ab	2.2	a	0.18	b	0.05	b	86	**
		VC	51		65		236		15			
Cu	Influent	Average	0.06	b	0.24	a	0.23	a	0.14	ab	55	**
		VC	105		35		61		53			
	R1	Average	0.06	b	0.13	a	0.11	ab	0.16	a	49	**
		VC	104		45		29		34			
	R2	Average	0.05	b	0.17	a	0.14	ab	0.15	ab	62	**
		VC	110		53		63		45			
Zn	Influent	Average	0.13	b	0.5	a	0.29	ab	0.24	b	58	**
		VC	100		45		62		46			
	R1	Average	0.1	b	0.28	a	0.27	a	0.23	a	65	**
		VC	91		43		78		47			
	R2	Average	0.1	b	0.34	a	0.18	ab	0.21	ab	69	**
		VC	75		51		99		50			

Units: mg L^{-1}. VC: variation coefficient in % of each phase. Different lowercase letters in the same line differ according to Tukey's test at 5%. F- F test. ** - Significant at 1% probability ($p<0.01$); ns - not significant ($p>0.05$); p-probability. VC*: coefficient of variation of each parameter for all phases.

Table S3. Average values of Ni and Co in the influent and effluent of the upflow anaerobic sludge blanket reactors (UASB), in series (R1 and R2) for the co-digestion of vinasse and molasses supplemented with filter cake, in phases 1, 2, 3 and 4.

Parameter	Samples		Phases									
			1		2		3		4		VC*	F
Co	Influent	Average	0.02	c	0.13	b	0.14	b	0.24	a	53	**
		VC	97		55		37		14			
	R1	Average	0.02	c	0.13	b	0.13	b	0.25	a	49	**
		VC	117		43		41		7			
	R2	Average	0.02	c	0.15	b	0.14	b	0.25	a	41	**
		VC	179		21		26		11			
Ni	Influent	Average	0.01	b	0.18	b	0.43	c	0.48	c	54	**
		VC	211		114		21		9			
	R1	Average	0.01	b	0.18	b	0.44	a	0.55	a	50	**
		VC	170		113		18		14			
	R2	Average	0.01	b	0.19	b	0.43	a	0.51	a	56	**
		VC	170		116		22		12			

Units: mg L^{-1}. VC: variation coefficient in % of each phase. Different lowercase letters in the same line differ according to Tukey's test at 5%. F- F test. ** - Significant at 1% probability (p<0.01); ns - not significant (p>0.05); p-probability. VC*: coefficient of variation of each parameter for all phases.

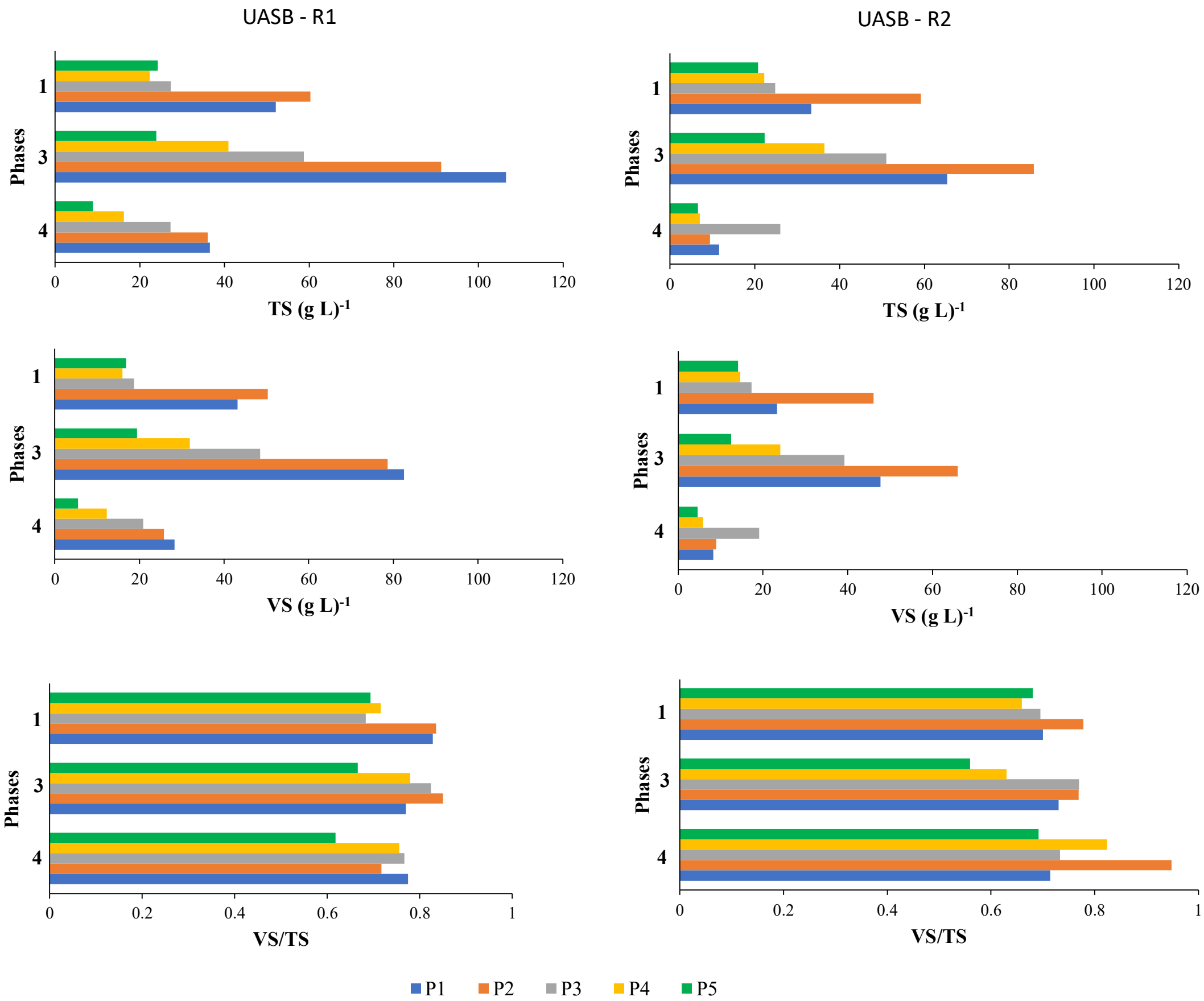

Figure S1. Total solids (TS) and volatile solids (VS) at the sludge collection points of the upflow anaerobic sludge reactors (UASB) in series, (R1 and R2) during the co-digestion of vinasse with molasses supplemented with filter cake in phases 1, 2, 3 and 4. P1: base of the reactor.

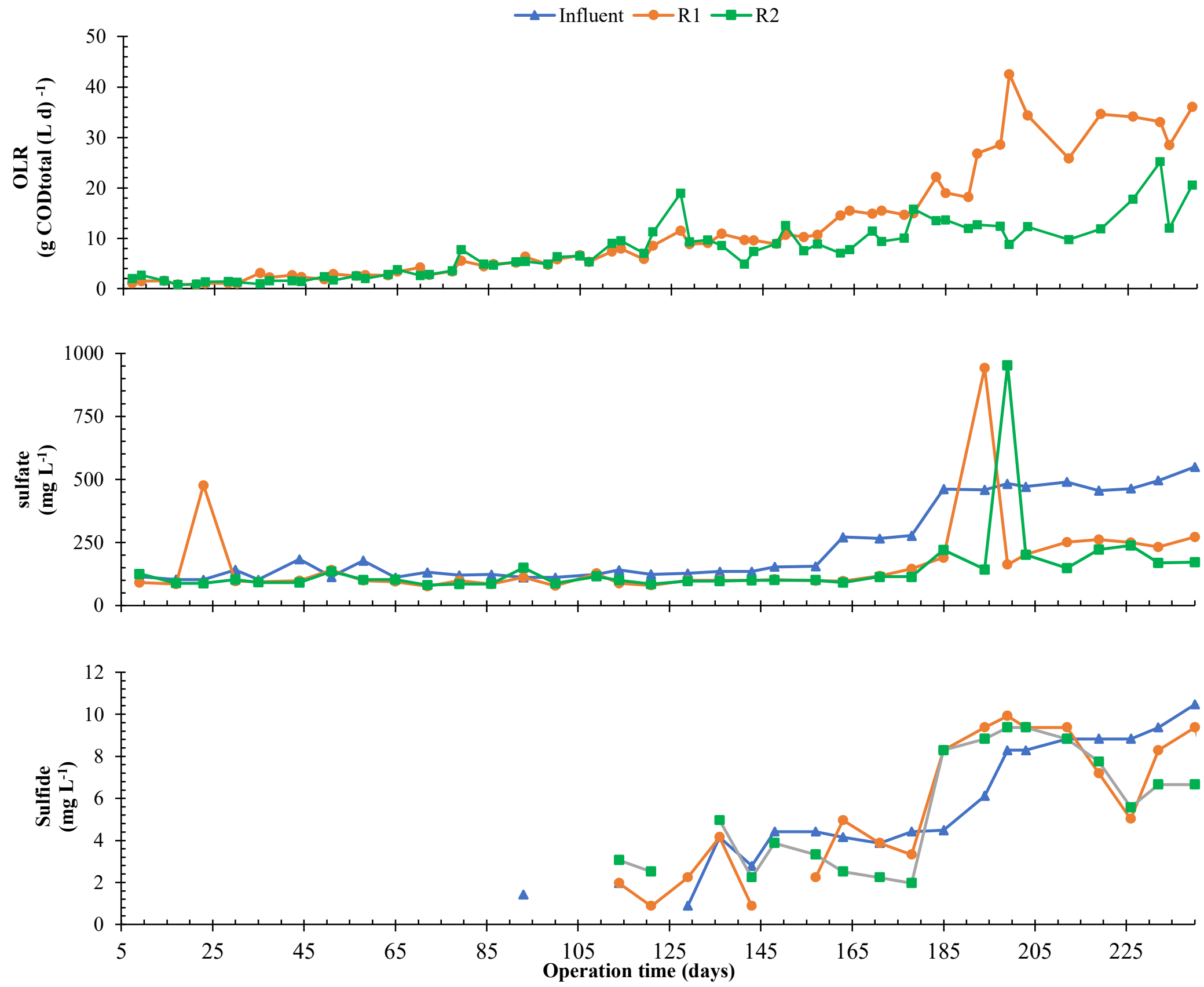

Figure S2. Values of sulfate and sulfide ions in the influent and effluents of the upflow anaerobic sludge blanket reactors (UASB), in series (R1 and R2), in the co-digestion of vinasse with molasses supplemented with filter cake, in phases 1, 2, 3, and 4.
